# 2,2′-[(Biphenyl-4,4′-di­yl)di(ethene-1,2-di­yl)]dibenzene­sulfonic acid–4-methyl­piperidine–water (1/2/2)

**DOI:** 10.1107/S1600536809026932

**Published:** 2009-07-18

**Authors:** Yu-Feng Li, Fang-Fang Jian

**Affiliations:** a Microscale Science Institute, Department of Chemistry and Chemical Engineering, Weifang University, Weifang 261061, People’s Republic of China; b Microscale Science Institute, Weifang University, Weifang 261061, People’s Republic of China

## Abstract

The title compound, C_28_H_22_O_6_S_2_·2C_6_H_13_N·2H_2_O, was prepared by the reaction of a Wittig reagent and 2-formyl­benzene­sulfonic acid. The main molecule lies about an inversion centre at the midpoint of the C—C bond between the inner benzene rings. The mol­ecular conformation is stabilized by intramolecular  hydrogen bonds. The crystal structure is further stabilized by O—H⋯O and N—H⋯O hydrogen-bonding inter­actions.

## Related literature

For the optical properties of ethyl­ene biphenyls, see: Song *et al.* (2003 ▶). For comparative bond lengths, see: Trueblood *et al.* (1982 ▶). 
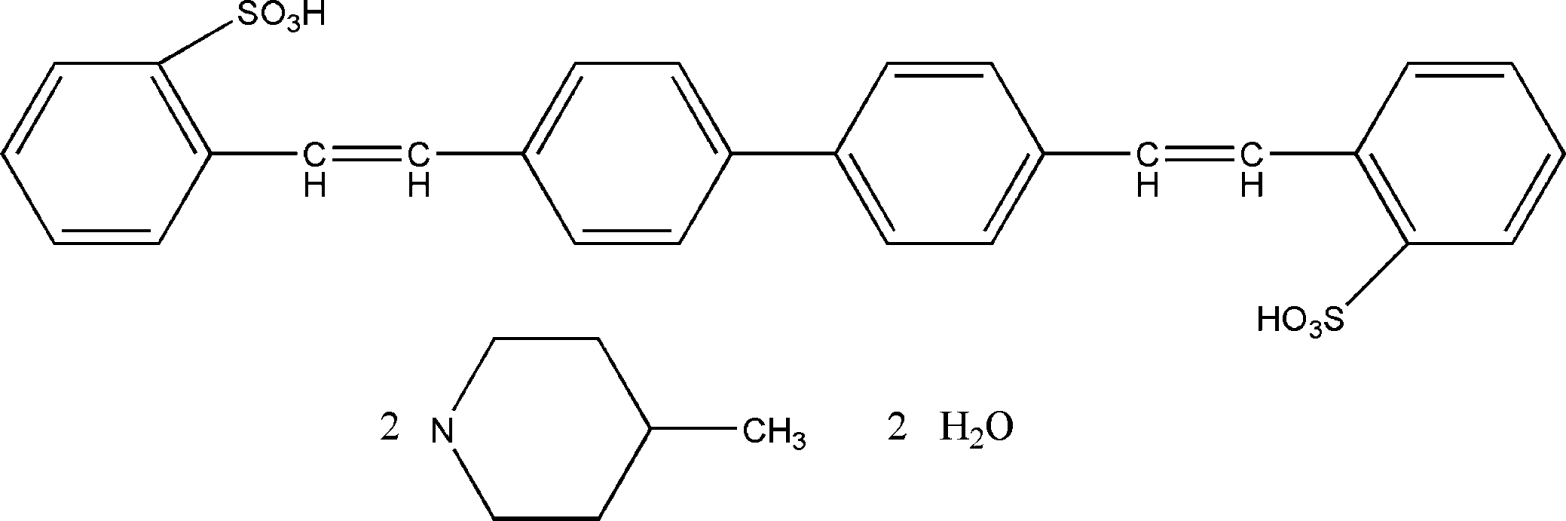


## Experimental

### 

#### Crystal data

C_28_H_22_O_6_S_2_·2(C_6_H_13_N)·2(H_2_O)
*M* *_r_* = 752.96
Monoclinic, 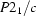

*a* = 14.852 (3) Å
*b* = 9.7240 (19) Å
*c* = 14.765 (3) Å
β = 109.76 (3)°
*V* = 2006.8 (7) Å^3^
*Z* = 2
Mo *K*α radiation
μ = 0.19 mm^−1^
*T* = 293 K
0.26 × 0.21 × 0.18 mm

#### Data collection

Bruker SMART CCD area-detector diffractometer
Absorption correction: none
4436 measured reflections
4264 independent reflections
1779 reflections with *I* > 2σ(*I*)
*R*_int_ = 0.026

#### Refinement

*R*[*F* ^2^ > 2σ(*F* ^2^)] = 0.060
*wR*(*F* ^2^) = 0.204
*S* = 1.01
4264 reflections
243 parameters
1 restraint
H atoms treated by a mixture of independent and constrained refinement
Δρ_max_ = 0.34 e Å^−3^
Δρ_min_ = −0.27 e Å^−3^

### 

Data collection: *SMART* (Bruker, 1997 ▶); cell refinement: *SAINT* (Bruker, 1997 ▶); data reduction: *SAINT*; program(s) used to solve structure: *SHELXS97* (Sheldrick, 2008 ▶); program(s) used to refine structure: *SHELXL97* (Sheldrick, 2008 ▶); molecular graphics: *SHELXTL* (Sheldrick, 2008 ▶); software used to prepare material for publication: *SHELXTL*.

## Supplementary Material

Crystal structure: contains datablocks global, I. DOI: 10.1107/S1600536809026932/at2832sup1.cif

Structure factors: contains datablocks I. DOI: 10.1107/S1600536809026932/at2832Isup2.hkl

Additional supplementary materials:  crystallographic information; 3D view; checkCIF report

## Figures and Tables

**Table 1:** Hydrogen-bond geometry (Å, °)

*D*—H⋯*A*	*D*—H	H⋯*A*	*D*⋯*A*	*D*—H⋯*A*
O1*W*—H1⋯O1	0.94 (6)	1.95 (6)	2.881 (5)	171 (6)
N2—H2*C*⋯O1*W*^i^	0.86	2.23	2.767 (4)	120
N2—H2*C*⋯O3^ii^	0.86	2.28	2.787 (5)	117
C2—H2*B*⋯O3	0.93	2.42	2.838 (5)	107
C7—H7*A*⋯O2	0.93	2.42	3.103 (5)	130

Symmetry codes: (i) 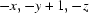; (ii) 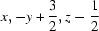.

**Fig. 1.**
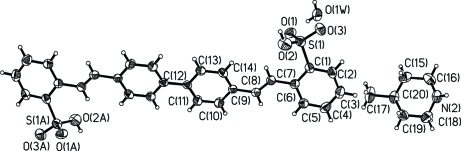
The structure of the title compound showing 30% probability displacement ellipsoids and the atom-numbering scheme.

## supplementary crystallographic information

### Comment

Ethylene biphenyl have received considerable attention in the literature. They are attractive from several points of view, such as the optics characteristic. (Song *et al.*, 2003). As part of our search for new ethylene biphenyl compounds we synthesized the title compound (I), and describe its structure here.

Main group of the title molecule in Fig. 1 has an inversion centre lied on the midpoint of the C—C bond between the inner benzene rings. The C7—C8 bond length of 1.326 (5)Å is comparable with C—C double bond [1.336 (2) Å] reported (Trueblood *et al.*,1982).

The molecular conformation is stabilized by C—H···O hydrogen bonds. The crystal structure is further stabilized by N—H···O hydrogen bonding interactions (Table 1).

### Experimental

A mixture of the Wittig-reagent (0.1 mol), and 2-formylbenzenesulfonic acid (0.2 mol) was stirred in refluxing 4-methylpiperidine (20 mL) for 4 h to afford the title compound (0.084 mol, yield 84%). Single crystals suitable for X-ray measurements were obtained by recrystallization from ethanol at room temperature.

### Refinement

The H atoms of the water molecule were found difference Fourier map and refined freely. The other atoms were fixed geometrically and allowed to ride on their attached atoms, with C—H = 0.93 - 0.97 Å O–H = 0.82 Å and with *U*_iso_(H)=1.2–1.5*U*_eq _(C, O).

### Crystal data

**Table:**

C_28_H_22_O_6_S_2_·2(C_6_H_13_N)·2(H_2_O)	*Z* = 2
*M**_r_* = 752.96	*F*(000) = 804
Monoclinic, *P*2_1_/*c*	*D*_x_ = 1.246 Mg m^−^^3^
Hall symbol: -P 2ybc	Mo *K*α radiation, λ = 0.71073 Å
*a* = 14.852 (3) Å	µ = 0.19 mm^−^^1^
*b* = 9.7240 (19) Å	*T* = 293 K
*c* = 14.765 (3) Å	Block, yellow
β = 109.76 (3)°	0.26 × 0.21 × 0.18 mm
*V* = 2006.8 (7) Å^3^	

### Data collection

**Table:**

Bruker SMART CCD area-detector diffractometer	1779 reflections with *I* > 2σ(*I*)
Radiation source: fine-focus sealed tube	*R*_int_ = 0.026
graphite	θ_max_ = 27.0°, θ_min_ = 1.5°
φ and ω scans	*h* = −17→17
4436 measured reflections	*k* = −11→0
4264 independent reflections	*l* = 0→17

### Refinement

**Table:**

Refinement on *F*^2^	Primary atom site location: structure-invariant direct methods
Least-squares matrix: full	Secondary atom site location: difference Fourier map
*R*[*F*^2^ > 2σ(*F*^2^)] = 0.060	Hydrogen site location: inferred from neighbouring sites
*wR*(*F*^2^) = 0.204	H atoms treated by a mixture of independent and constrained refinement
*S* = 1.01	*w* = 1/[σ^2^(*F*_o_^2^) + (0.0935*P*)^2^ + 0.2207*P*] where *P* = (*F*_o_^2^ + 2*F*_c_^2^)/3
4264 reflections	(Δ/σ)_max_ < 0.001
243 parameters	Δρ_max_ = 0.34 e Å^−^^3^
1 restraint	Δρ_min_ = −0.27 e Å^−^^3^

### Special details

**Table:**

Geometry. All e.s.d.'s (except the e.s.d. in the dihedral angle between two l.s. planes) are estimated using the full covariance matrix. The cell e.s.d.'s are taken into account individually in the estimation of e.s.d.'s in distances, angles and torsion angles; correlations between e.s.d.'s in cell parameters are only used when they are defined by crystal symmetry. An approximate (isotropic) treatment of cell e.s.d.'s is used for estimating e.s.d.'s involving l.s. planes.
Refinement. Refinement of *F*^2^ against ALL reflections. The weighted *R*-factor *wR* and goodness of fit *S* are based on *F*^2^, conventional *R*-factors *R* are based on *F*, with *F* set to zero for negative *F*^2^. The threshold expression of *F*^2^ > σ(*F*^2^) is used only for calculating *R*-factors(gt) *etc*. and is not relevant to the choice of reflections for refinement. *R*-factors based on *F*^2^ are statistically about twice as large as those based on *F*, and *R*- factors based on ALL data will be even larger.

### Fractional atomic coordinates and isotropic or equivalent isotropic displacement parameters (Å^2^)

**Table:**

	*x*	*y*	*z*	*U*_iso_*/*U*_eq_	
S1	0.16287 (7)	0.72108 (11)	0.45278 (7)	0.0650 (4)	
O1	0.11710 (19)	0.6177 (3)	0.49284 (19)	0.0777 (8)	
O2	0.2213 (2)	0.8160 (3)	0.5239 (2)	0.0963 (10)	
H2A	0.1872	0.8610	0.5464	0.144*	
O3	0.0962 (2)	0.7896 (3)	0.3708 (2)	0.0946 (10)	
C1	0.2421 (2)	0.6277 (4)	0.4076 (2)	0.0547 (9)	
C2	0.2274 (3)	0.6354 (4)	0.3096 (3)	0.0685 (11)	
H2B	0.1789	0.6909	0.2704	0.082*	
C3	0.2841 (3)	0.5616 (5)	0.2699 (3)	0.0813 (13)	
H3B	0.2744	0.5689	0.2045	0.098*	
C4	0.3556 (3)	0.4763 (5)	0.3275 (3)	0.0797 (13)	
H4A	0.3918	0.4229	0.3004	0.096*	
C5	0.3726 (3)	0.4712 (4)	0.4253 (3)	0.0673 (11)	
H5A	0.4222	0.4164	0.4637	0.081*	
C6	0.3174 (2)	0.5464 (4)	0.4686 (2)	0.0549 (9)	
C7	0.3396 (2)	0.5464 (4)	0.5737 (2)	0.0572 (10)	
H7A	0.3073	0.6108	0.5979	0.069*	
C8	0.4005 (2)	0.4653 (4)	0.6378 (3)	0.0593 (10)	
H8A	0.4296	0.3958	0.6143	0.071*	
C9	0.4261 (2)	0.4755 (4)	0.7428 (2)	0.0527 (9)	
C10	0.4910 (3)	0.3842 (5)	0.8026 (3)	0.0808 (13)	
H10A	0.5164	0.3147	0.7752	0.097*	
C11	0.5191 (3)	0.3931 (5)	0.9011 (3)	0.0785 (13)	
H11A	0.5627	0.3292	0.9382	0.094*	
C12	0.4847 (2)	0.4936 (4)	0.9467 (2)	0.0502 (9)	
C13	0.4184 (3)	0.5858 (4)	0.8868 (3)	0.0680 (11)	
H13A	0.3927	0.6548	0.9142	0.082*	
C14	0.3906 (3)	0.5760 (4)	0.7881 (3)	0.0679 (11)	
H14A	0.3466	0.6390	0.7506	0.081*	
C17	0.1822 (5)	0.2643 (6)	0.1068 (3)	0.131 (2)	
H17A	0.1298	0.2262	0.1227	0.196*	
H17B	0.1970	0.3543	0.1346	0.196*	
H17C	0.2372	0.2060	0.1317	0.196*	
C15	0.0653 (3)	0.3594 (5)	−0.0460 (4)	0.0913 (15)	
H15A	0.0749	0.4489	−0.0152	0.110*	
H15B	0.0127	0.3150	−0.0327	0.110*	
C16	0.0386 (3)	0.3788 (6)	−0.1540 (4)	0.0992 (16)	
H16A	0.0216	0.2909	−0.1863	0.119*	
H16B	−0.0163	0.4394	−0.1773	0.119*	
N2	0.1191 (3)	0.4378 (4)	−0.1755 (2)	0.0859 (11)	
H2C	0.1167	0.5144	−0.2053	0.103*	
C18	0.2061 (4)	0.3503 (5)	−0.1398 (3)	0.0917 (14)	
H18A	0.2582	0.3927	−0.1554	0.110*	
H18B	0.1935	0.2609	−0.1706	0.110*	
C19	0.2335 (3)	0.3339 (5)	−0.0337 (3)	0.0815 (13)	
H19A	0.2516	0.4229	−0.0033	0.098*	
H19B	0.2890	0.2741	−0.0111	0.098*	
C20	0.1544 (3)	0.2747 (4)	−0.0032 (3)	0.0777 (12)	
H20A	0.1410	0.1815	−0.0297	0.093*	
O1W	−0.0458 (3)	0.4739 (4)	0.3645 (2)	0.0939 (11)	
H2	−0.082 (3)	0.537 (4)	0.380 (3)	0.074 (16)*	
H1	0.009 (3)	0.512 (7)	0.410 (4)	0.17 (3)*	

### Atomic displacement parameters (Å^2^)

**Table:**

	*U*^11^	*U*^22^	*U*^33^	*U*^12^	*U*^13^	*U*^23^
S1	0.0645 (6)	0.0621 (6)	0.0658 (6)	0.0026 (6)	0.0188 (5)	0.0124 (6)
O1	0.0773 (18)	0.080 (2)	0.090 (2)	0.0010 (15)	0.0466 (16)	0.0174 (16)
O2	0.101 (2)	0.0657 (19)	0.117 (2)	0.0039 (17)	0.0292 (19)	−0.0220 (18)
O3	0.090 (2)	0.108 (2)	0.0765 (19)	0.0237 (19)	0.0168 (16)	0.0307 (18)
C1	0.048 (2)	0.056 (2)	0.054 (2)	−0.0167 (18)	0.0110 (17)	0.0052 (18)
C2	0.061 (2)	0.084 (3)	0.054 (2)	−0.018 (2)	0.010 (2)	0.009 (2)
C3	0.085 (3)	0.105 (4)	0.055 (3)	−0.030 (3)	0.025 (2)	−0.006 (3)
C4	0.077 (3)	0.096 (3)	0.076 (3)	−0.024 (3)	0.039 (2)	−0.019 (3)
C5	0.055 (2)	0.085 (3)	0.064 (3)	−0.007 (2)	0.022 (2)	−0.001 (2)
C6	0.050 (2)	0.060 (2)	0.058 (2)	−0.0139 (18)	0.0220 (18)	0.0004 (18)
C7	0.056 (2)	0.061 (2)	0.055 (2)	0.0001 (19)	0.0205 (19)	0.0048 (18)
C8	0.053 (2)	0.067 (3)	0.060 (2)	0.0047 (19)	0.0217 (18)	0.003 (2)
C9	0.048 (2)	0.059 (2)	0.053 (2)	0.0041 (18)	0.0196 (17)	0.0057 (19)
C10	0.094 (3)	0.086 (3)	0.063 (3)	0.044 (3)	0.027 (2)	0.006 (2)
C11	0.091 (3)	0.084 (3)	0.057 (3)	0.045 (3)	0.021 (2)	0.012 (2)
C12	0.0452 (19)	0.053 (2)	0.0534 (19)	0.0041 (18)	0.0180 (17)	0.0099 (19)
C13	0.070 (3)	0.070 (3)	0.061 (3)	0.028 (2)	0.017 (2)	0.001 (2)
C14	0.063 (2)	0.073 (3)	0.059 (3)	0.025 (2)	0.009 (2)	0.011 (2)
C17	0.210 (7)	0.108 (4)	0.075 (3)	0.012 (4)	0.051 (4)	0.012 (3)
C15	0.091 (3)	0.081 (3)	0.123 (4)	−0.013 (3)	0.065 (3)	0.010 (3)
C16	0.075 (3)	0.100 (4)	0.100 (4)	−0.001 (3)	0.001 (3)	0.000 (3)
N2	0.106 (3)	0.077 (2)	0.075 (2)	−0.001 (2)	0.032 (2)	0.020 (2)
C18	0.108 (4)	0.088 (3)	0.096 (4)	0.005 (3)	0.057 (3)	−0.003 (3)
C19	0.075 (3)	0.077 (3)	0.086 (3)	0.016 (2)	0.019 (2)	−0.010 (2)
C20	0.114 (4)	0.055 (2)	0.066 (3)	−0.001 (3)	0.033 (2)	0.001 (2)
O1W	0.108 (3)	0.106 (3)	0.068 (2)	0.000 (2)	0.030 (2)	0.0108 (19)

### Geometric parameters (Å, °)

**Table:**

S1—O3	1.442 (3)	C12—C12^i^	1.488 (6)
S1—O2	1.446 (3)	C13—C14	1.377 (5)
S1—O1	1.447 (3)	C13—H13A	0.9300
S1—C1	1.784 (4)	C14—H14A	0.9300
O2—H2A	0.8200	C17—C20	1.537 (5)
C1—C2	1.392 (5)	C17—H17A	0.9600
C1—C6	1.417 (5)	C17—H17B	0.9600
C2—C3	1.378 (6)	C17—H17C	0.9600
C2—H2B	0.9300	C15—C20	1.504 (6)
C3—C4	1.389 (6)	C15—C16	1.519 (6)
C3—H3B	0.9300	C15—H15A	0.9700
C4—C5	1.379 (5)	C15—H15B	0.9700
C4—H4A	0.9300	C16—N2	1.455 (6)
C5—C6	1.404 (5)	C16—H16A	0.9700
C5—H5A	0.9300	C16—H16B	0.9700
C6—C7	1.473 (5)	N2—C18	1.487 (5)
C7—C8	1.326 (5)	N2—H2C	0.8600
C7—H7A	0.9300	C18—C19	1.488 (5)
C8—C9	1.469 (5)	C18—H18A	0.9700
C8—H8A	0.9300	C18—H18B	0.9700
C9—C14	1.384 (5)	C19—C20	1.508 (6)
C9—C10	1.387 (5)	C19—H19A	0.9700
C10—C11	1.373 (5)	C19—H19B	0.9700
C10—H10A	0.9300	C20—H20A	0.9800
C11—C12	1.380 (5)	O1W—H2	0.90 (4)
C11—H11A	0.9300	O1W—H1	0.94 (6)
C12—C13	1.402 (5)		
			
O3—S1—O2	112.82 (19)	C12—C13—H13A	119.4
O3—S1—O1	112.34 (19)	C13—C14—C9	122.2 (3)
O2—S1—O1	113.35 (19)	C13—C14—H14A	118.9
O3—S1—C1	105.87 (18)	C9—C14—H14A	118.9
O2—S1—C1	106.64 (17)	C20—C17—H17A	109.5
O1—S1—C1	105.03 (16)	C20—C17—H17B	109.5
S1—O2—H2A	109.5	H17A—C17—H17B	109.5
C2—C1—C6	120.2 (4)	C20—C17—H17C	109.5
C2—C1—S1	118.1 (3)	H17A—C17—H17C	109.5
C6—C1—S1	121.7 (3)	H17B—C17—H17C	109.5
C3—C2—C1	120.8 (4)	C20—C15—C16	113.0 (4)
C3—C2—H2B	119.6	C20—C15—H15A	109.0
C1—C2—H2B	119.6	C16—C15—H15A	109.0
C2—C3—C4	120.0 (4)	C20—C15—H15B	109.0
C2—C3—H3B	120.0	C16—C15—H15B	109.0
C4—C3—H3B	120.0	H15A—C15—H15B	107.8
C5—C4—C3	119.6 (4)	N2—C16—C15	109.6 (4)
C5—C4—H4A	120.2	N2—C16—H16A	109.8
C3—C4—H4A	120.2	C15—C16—H16A	109.8
C4—C5—C6	122.0 (4)	N2—C16—H16B	109.8
C4—C5—H5A	119.0	C15—C16—H16B	109.8
C6—C5—H5A	119.0	H16A—C16—H16B	108.2
C5—C6—C1	117.3 (3)	C16—N2—C18	112.2 (4)
C5—C6—C7	121.5 (3)	C16—N2—H2C	123.9
C1—C6—C7	121.2 (3)	C18—N2—H2C	123.9
C8—C7—C6	127.4 (4)	N2—C18—C19	109.3 (3)
C8—C7—H7A	116.3	N2—C18—H18A	109.8
C6—C7—H7A	116.3	C19—C18—H18A	109.8
C7—C8—C9	125.7 (4)	N2—C18—H18B	109.8
C7—C8—H8A	117.2	C19—C18—H18B	109.8
C9—C8—H8A	117.2	H18A—C18—H18B	108.3
C14—C9—C10	116.1 (3)	C18—C19—C20	113.1 (4)
C14—C9—C8	123.5 (3)	C18—C19—H19A	109.0
C10—C9—C8	120.3 (3)	C20—C19—H19A	109.0
C11—C10—C9	122.0 (4)	C18—C19—H19B	109.0
C11—C10—H10A	119.0	C20—C19—H19B	109.0
C9—C10—H10A	119.0	H19A—C19—H19B	107.8
C10—C11—C12	122.1 (3)	C15—C20—C19	109.2 (3)
C10—C11—H11A	118.9	C15—C20—C17	111.4 (4)
C12—C11—H11A	118.9	C19—C20—C17	112.4 (4)
C11—C12—C13	116.2 (3)	C15—C20—H20A	107.9
C11—C12—C12^i^	123.0 (4)	C19—C20—H20A	107.9
C13—C12—C12^i^	120.8 (4)	C17—C20—H20A	107.9
C14—C13—C12	121.3 (3)	H2—O1W—H1	90 (3)
C14—C13—H13A	119.4		

Symmetry codes: (i) −*x*+1, −*y*+1, −*z*+2.

### Hydrogen-bond geometry (Å, °)

**Table:**

*D*—H···*A*	*D*—H	H···*A*	*D*···*A*	*D*—H···*A*
O1W—H1···O1	0.94 (6)	1.95 (6)	2.881 (5)	171 (6)
N2—H2C···O1W^ii^	0.86	2.23	2.767 (4)	120
N2—H2C···O3^iii^	0.86	2.28	2.787 (5)	117
C2—H2B···O3	0.93	2.42	2.838 (5)	107
C7—H7A···O2	0.93	2.42	3.103 (5)	130

Symmetry codes: (ii) −*x*, −*y*+1, −*z*; (iii) *x*, −*y*+3/2, *z*−1/2.

## References

[bb1] Bruker (1997). *SMART* and *SAINT* Bruker AXS Inc., Madison, Wisconsin, USA.

[bb2] Sheldrick, G. M. (2008). *Acta Cryst.* A**64**, 112–122. 10.1107/S0108767307043930 18156677

[bb3] Song, H. C., Xu, X. H. & Liu, G. R. (2003). *Chin. Chem. Res.* **14**, 1–5.

[bb4] Trueblood, K., Mirsky, K., Maverick, E., Knobler, C. & Grossenbacher, L. (1982). *Acta Cryst.* B**38**, 2428–2435.

